# Cervical screening: A complete and patient centred approach

**DOI:** 10.4102/safp.v63i1.5372

**Published:** 2021-10-27

**Authors:** Claire van Deventer

**Affiliations:** 1 Department of Family Medicine and Primary care, Faculty of Health Sciences, University of the Witwatersrand, Johannesburg, South Africa

**Keywords:** cervical screening, HPV, VIA, step-by-step procedure

## Abstract

Cervical screening is an important intervention in the detection and management of early cancer. The global situation regarding cervical cancer is briefly discussed and a family physician’s approach to the procedure and the response to laboratory findings is documented.

## Introduction

In a commitment to the global eradication of cervical cancer, the World Health Organization (WHO) launched an initiative in November 2020, involving 194 countries. The aim is to reduce new cases of cancer by 40% and to save 5 million lives. To achieve this aim, objectives are to vaccinate 90% of young girls against human papillomavirus (HPV), screen 70% of women 35 years and older and to appropriately treat 90% of women who have been diagnosed with cervical cancer.^1^ South Africa is one of the signatories to this programme.

The estimated age-standardised incidence rate (ASIR) of cervical cancer was 13.1 women-years per 100 000 globally in 2018, making it a serious health issue worthy of urgent intervention.^2^ Rates however range widely between higher and lower income countries. South Africa has an ASIR of 43.1 per 100 000 and an age standardised mortality rate (ASMR) of 19 per 100 000 as compared with China (10 per 100 000 and 4 per 100 000), Brazil (12 per 100 000 and 5 per 100 000) and the United Kingdom (4 per 100 000 and < 1.5 per 100 000)^2^ to mention a few countries on different continents. This is also the second most common cancer in women in South Africa. In 2018 there were 12 983 new cases diagnosed in the country.^2^

High risk factors for HPV appear to be infection with HIV and other sexually transmitted illness (STIs), increased numbers of sexual partners, early sexual debut (<16 years) and gender-based violence (GBV).^3,4^

Cervical pre-cancer has a very slow progression; there is therefore a window of opportunity, even in low-resourced countries to discover and manage it in time. The South African guidelines on cervical screening (2019) suggested a minimum of three cervical smears per lifetime, once a decade from age 30, for patients with no comorbidities such as HIV and no abnormal smears.^5,6^

The purpose of this continuing professional development (CPD) article is to demonstrate the steps in cervical screening and to inform the reader of the optimal management of results.

The two most available screening methods in South Africa are the cervical smear with (1) an Ayre’s spatula and (2) liquid-based cytology using a brush. The public service uses the former method, or a hybrid of a brush and slides, because of the need for cost containment. The second technique is the preferred method in private practice. There is however a significant difference in results between the two methods, with the liquid-based cytology^7,8,9^ being superior to the traditional method in terms of sensitivity.

Human papilloma virus testing could be performed at the same time or alone, if liquid-based cytology is used. It is the South African Society for Obstetricians and Gynaecologist’s (SASOGs) opinion that ‘HPV-based primary screening is more sensitive to detect pre-cancer and cancer and also has a better negative predictive value … than cytology’.^10^

### Factors to consider before performing cervical smear

The patient should preferably not be menstruating. If it is possible she should be informed that there should be no insertion of creams or medications a few days before the test. The use of tampons is also discouraged.

**FIGURE 1:**
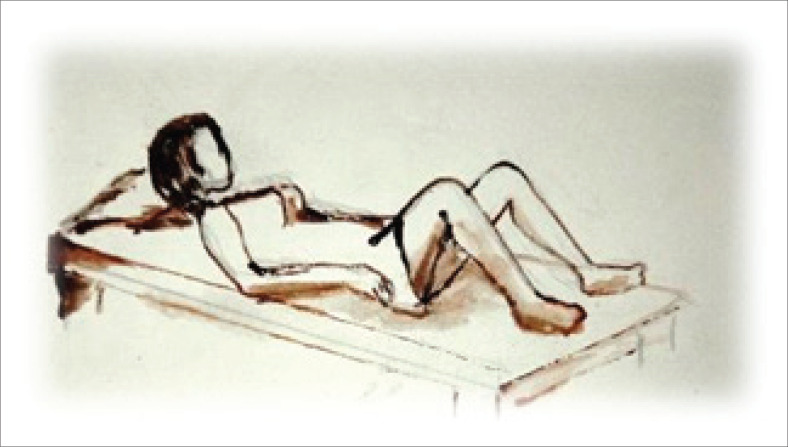
Lithotomy position.

**FIGURE 2:**
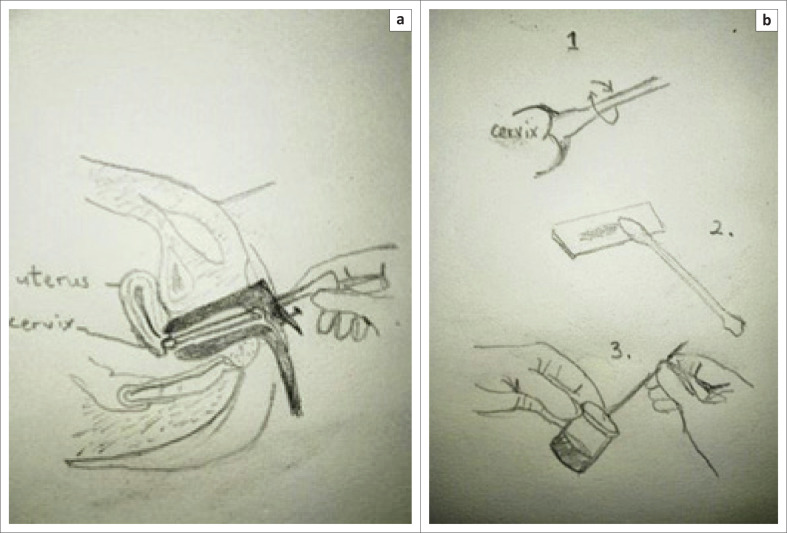
Steps for taking a cervical smear. a) Inserting speculum and spatula; (b) 1. 360 degree smear; 2. Apply Ayres spatula to slide or 3. break off end of the brush into cytology fluid.

**FIGURE 3:**
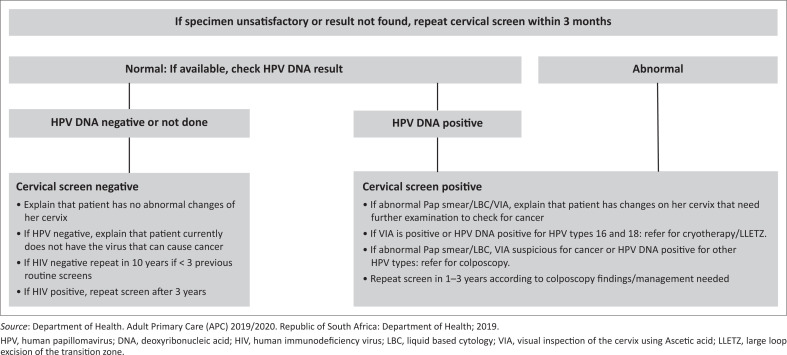
A guide to the management of cervical smear results.

In a short history before the procedure, check with the patient about the possibility of pregnancy, allergies (e.g. latex) and unusual symptoms such as spotting or discharges:

- The following equipment should be ready: The correct size speculum, Ayre’s spatula or cervical brush, cotton wool swabs, labelled slides and fixative spray for traditional screening and liquid-based bottle if brush is being used.
- Gain informed consent from the patient and explain in a clear manner what the process involves. During the procedure explain to the patient what you are doing.
- Allow the patient to pass urine before the test.
- Give the patient privacy to undress and make a gown or sheet available for her to use.
- Consider the use of a chaperone.
- Let the patient lie in the lithotomy position and encourage her to relax and allow her knees to fall outwards. (Figure 1)
- Visually examine the genital area for any signs of disease.
- Warm the speculum in warm water or use warmed lubricant.
- While explaining what you are doing, separate the labia and insert the speculum gently into the vagina; blades closed, angling downward, using little or no K-Y Jelly. If K-Y Jelly is used it may contaminate the specimen and should not be applied to the tip of the speculum.
- Once inserted, rotate the speculum to 90° and open the blades until a good view of the cervix is obtained.
- Tighten the locking screw to keep the speculum in place.
- Carefully clean any blood or discharge if present, from the cervix with a cotton wool swab.
- Visualise the cervix clearly and inspect for ulcers, cervicitis and tumours.
- Insert the spatula or brush into the endocervical canal and rotate 360 degree a few times.
- Remove the spatula/brush without touching the sides of the speculum.
- Apply the smear onto the glass slide within 30 seconds, with light sweeping movements to get a thin layer and spray immediately with fixative, holding the bottle about 30 cm away or insert the brush into the liquid-based container and break it off and seal.
- Remove the speculum (Figure 2) by unlocking the screw and partly closing the blades.
- A bimanual pelvic exam could now be performed if indicated.
- Allow the patient to get dressed and provide paper towels to clean herself.
- Allow her to voice any concerns or questions at this stage.
- Fill in the appropriate forms. Hormonal treatment and the appearance of the cervix are useful to note on the form as well. Human Papillomavirus should be requested if liquid-based cytology is used.
- Ensure that the patient’s contact details are correct in order to contact her. Inform her that she may phone for the results if she has heard nothing within a certain period of time. (Figure 3)

## Conclusion

A truly holistic approach towards a patient is what should differentiate a family physician from any other practitioner. Acknowledging the patient’s autonomy, allowing her to verbalise her expectations and fears and responding to verbal and non-verbal cues during the process are all aspects of a family physician’s practice. This is as true of a procedure such as a cervical screen as it is of a consultation.

Together with this, clinical competence and the latest evidence should be employed to reach the best conclusion for each patient’s health.
